# Supplementary material for: Multistage Molecular Simulations, Design, Synthesis, and Anticonvulsant Evaluation of 2-(Isoindolin-2-yl) Esters of Aromatic Amino Acids Targeting GABAA Receptors via π-π Stacking
Source: Int J Mol Sci. 2025 Jul 15;26(14):6780. doi: 10.3390/ijms26146780 (PMC12295343; doi:10.3390/ijms26146780)
Supplement: Supplementary file 1 [file ijms-26-06780-s001.zip › ijms-3716569-supplementary.pdf]

<u>**Supporting Information**</u>

Multi-Stage Molecular Simulation Design, Synthesis, and Anticonvulsant Evaluation of 2-(Isoindolin-2-yl) Esters of Aromatic Amino Acids Targeting $GABA_A$ Receptors via π–π Interactions

Santiago González-Periañez† [1,2], Fabiola Hernández-Rosas† [3,4], Carlos Alberto López-Rosas[5], Fernando Rafael Ramos-Morales[1,5,7], Jorge Zurutuza-Lorméndez[6], Rosa Virginia García-Rodríguez[5,7], José Luís Olivares-Romero[8], Rodrigo Rafael Ramos-Hernández[7], Ivette Bravo-Espinoza[7], Abraham Vidal-Limon[8*], Tushar Janardan Pawar[8,9*]

1 Centro de Investigaciones Biomédicas, Doctorado en Ciencias Biomédicas, Universidad Veracruzana, Xalapa 91190, México. santiagonzalez@uv.mx (S.G.-P).
2 Facultad de Bioanálisis, Universidad Veracruzana, Calle Médicos y Odontólogos s/n, Unidad del Bosque, C.P. 91010. Xalapa-Enríquez, Veracruz, México.
3 Centro de Investigación, Universidad Anáhuac Querétaro, El Marqués, Querétaro 76246, México. fabiola.hernandezro@anahuac.mx (F.H.-R.)
4 Facultad de Química, Universidad Autónoma de Querétaro, Querétaro 76010, México. (F.H.-R)
5 Facultad de Química Biológica Farmacéutica, Universidad Veracruzana, Gonzalo Aguirre Beltrán s/n. Col. Centro, C.P. 91000, Xalapa, Veracruz, México. carloslopez02@uv.mx (C.A.L-R); ivbravo@uv.mx (I.B-E.); rodrigoqfb@gmail.com (R.R.R-H).
6 Centro de Salud Urbano José A. Maraboto Carreón, Servicios de Salud de Veracruz, Santiago Bonilla No 85, Col. Obrero Campesino, CP. 91020, Xalapa, Veracruz, México. jorzurutuza@uv.mx (J.I.Z.-L).
7 Instituto de Química Aplicada, Universidad Veracruzana, Luis Castelazo Ayala s/n, Col. Industrial Animas, 91190, Xalapa-Enríquez, Veracruz, México; framos@uv.mx, (F.R.R.-M), rosagarcia02@uv.mx (R.V.G.-R.).
8 Red de Estudios Moleculares Avanzados, Instituto de Ecología A.C. (INECOL), Carretera Antigua a Coatepec 351, CP. 91073, El Haya, Xalapa, Veracruz, México; jose.olivares@inecol.mx (J.L.O.-R), tushar.janardan@inecol.mx (T.J.P.), abraham.vidal@inecol.mx (A.V.L.).
9 Unidad de Desarrollo e Investigación en Bioterapeúticos (UDIBI), Escuela Nacional de Ciencias Biológicas, Instituto Politécnico Nacional, 11340 Mexico City, Mexico.
* Correspondence: abraham.vidal@inecol.mx (A.V.L.); tushar.janardan@inecol.mx (T.J.P.)
† S.G.-P and F.H.R contributed equally to this paper to be considered as first authors.

INDEX

1 General Methods ... 3
2 Synthesis and Characterization ... 5
2.1 Synthesis of methyl (*S*)-(1*H*-indol-3-yl)-2-(isoindolin-2-yl) propanoate (1) ... 5
2.2 Synthesis of methyl (*S*)-3-(4-hydroxyphenyl)-2-(isoindolin-2-yl) propanoate (2) ... 5
3 Biological assay ... 7
4 ADMET Profiling of Selected Schiff Base Derivatives ... 8
5 Plots, Statistical Analysis and Visualizations ... 14
5.1 Descriptive Statistics ... 14
6 Structure-Activity Relationship (SAR) ... 22
6.1 Aromatic Preference and Workflow ... 22
6.1.1 Library Design: Sixteen Isoindoline Esters Derived from Natural Amino Acids ... 22
6.1.2 Grouping Compounds by Side Chain Type ... 22
6.1.3 Summary of Core Properties ... 23
6.2 Physicochemical and ADMET Filtering ... 24
6.2.1 Physicochemical Space and CNS Compatibility ... 24
6.2.2 Hydrogen Bonding Profiles ... 25
6.2.3 ADMET Prediction Results ... 25
6.2.4 Conclusion of Filtering Step ... 25
6.3 Molecular Docking & π–π Interaction Profile ... 26
6.3.1 Docking Score Comparison and Prioritization ... 26
6.3.2 Interaction Mapping and Pose Analysis ... 26
6.3.3 Receptor Environment and Aromatic Selectivity ... 26
6.3.4 Conclusion ... 26
6.4 Binding Energy Landscape (Metadynamics) ... 27
6.4.1 Objective and Rationale ... 27
6.4.2 Simulation Protocol ... 27
6.4.3 Free Energy Profiles ... 28
6.4.4 Interpretation of Binding Modes ... 28
6.4.5 Summary ... 28
6.5 Receptor-Specific Interpretation ... 29
6.5.1 $GABA_A$ Receptor Binding Site Characteristics ... 29
6.5.2 Implications for Ligand Design ... 29
6.5.3 Mimicry of Classical Benzodiazepines ... 29

6.5.4 Conclusion ... 30
6.6 Comparative SAR Insights and Outliers ... 30
6.6.1 Ranking and Performance Clustering ... 30
6.6.2 Outlier Analysis ... 30
6.6.3 Non-Aromatic Class Performance ... 31
6.6.4 SAR Summary Table Reference ... 31
6.7 Design Implications and Next-Generation Analogs ... 31
6.7.1 Key Design Principles ... 31
6.7.2 Recommended Modifications ... 31
6.7.3 Broader Application ... 32
6.7.4 Conclusion ... 32
6.8 SAR Conclusion ... 32
7 NMR and MS Specters ... 34
8 References ... 40

1 General Methods

Chemicals and reagents, including solvents, were purchased from Sigma-Aldrich and used without further purification. Glassware was oven-dried prior to use, and heating reactions were conducted in a heating blanket.

Thin-layer chromatography (TLC) was performed on pre-coated aluminum plates of silica gel 60 F254 (0.25 mm, E. Merck). Plates were visualized under a short-wave UV lamp and by heating after dipping in a ninhydrin solution. Column chromatography was conducted using silica gel (100–200 mesh and 230–400 mesh), with eluents chosen based on polarity correlated to TLC mobility.

The Isolera One Biotage equipment was used to purify the compounds with a AcOEt/Hx 1:1 mobile phase and Biotage SNAP Cartridge, silica, 10 g.

NMR spectroscopy was conducted using an Agilent technologies NMR 500/54 premium shielded spectrometer with deuterated chloroform ($CDCl_3$-*d*, 99.8%) as the solvent. Chemical shifts (δ) are

reported in parts per million (ppm) relative to solvent peaks. Data for ^{1}H NMR are reported as follows: chemical shift (δ, ppm), multiplicity (s: singlet, d: doublet, dd: doublet of doublets, t: triplet, q: quartet, m: multiplet), coupling constant (J in Hz), integration, and assigned protons.

Mass spectra were a were obtained by electron impact ionization at 70 eV using a mass spectrometer Agilent Technologies model 5975 inert XL, and the raw data were processed using the associated software. The spectra provided high-resolution mass measurements, aiding in the confirmation of molecular structures.

2 Synthesis and Characterization

2.1 Synthesis of methyl (*S*)-(1*H*-indol-3-yl)-2-(isoindolin-2-yl) propanoate (1)

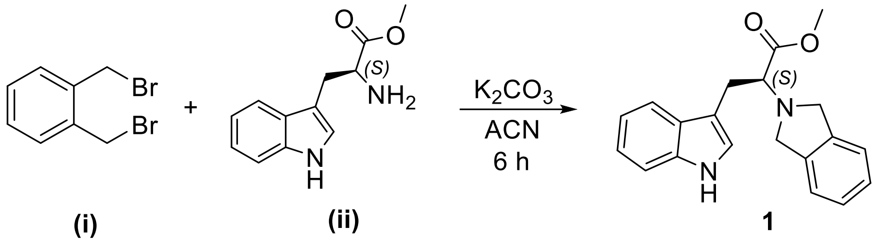

α, α'-dibromo-*o*-xylene **(i)** (1 g, 3.78 mmol) with *L*-tryptophan methyl ester **(ii)** (0.96 g, 3.78 mmol) and of potassium carbonate (1.3 g, 9.45 mmol) on 50 mL of acetonitrile was added and refluxed at 85 °C for 6 h. Reaction mixture was filtered and concentrated and purified by Isolera One Biotage device with a AcOEt/Hx 1:1 mobile phase and Biotage SNAP Cartridge, silica, 10 g to afford isoindoline compound **1** as brown solid (0.72 g; 75%).

^{1}H NMR (500 MHz, CDCl$_3$-*d*): δ 7.20 (s, 4H), 7.09 – 7.04 (m, 2H), 6.71 – 6.65 (m, 2H), 4.29 – 4.21 (m, 3H), 4.18 – 4.10 (m, 2H), 3.75 (dd, J = 8.7, 6.6 Hz, 1H), 3.61 (s, 3H), 3.12 – 3.03 (m, 2H).

^{13}C NMR (126 MHz, CDCl$_3$-*d*): δ 173.09, 139.35, 136.13, 127.42, 126.85, 123.12, 122.69, 122.43, 121.95, 119.35, 118.54, 111.51, 111.24, 77.34, 77.08, 76.83, 65.97, 55.68, 51.44, 27.00.

MS (ESI) (m/z): $[M + H]^+$ calculated for 320.2

2.2 Synthesis of methyl (*S*)-3-(4-hydroxyphenyl)-2-(isoindolin-2-yl) propanoate (2)

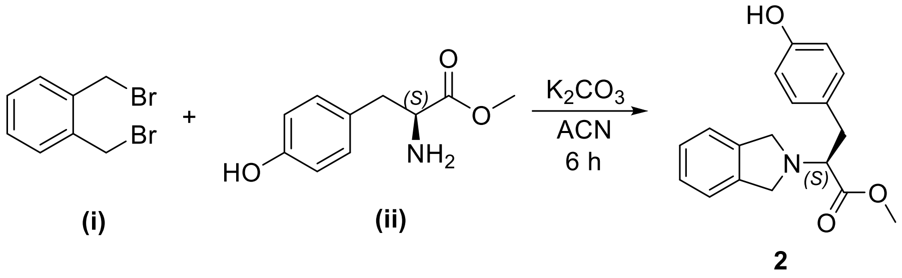

α, α'-dibromo-*o*-xylene **(i)** (0.5 g, 1.90 mmol) with *L*-tyrosine methyl ester **(ii)** (0.44 g, 1.90 mmol) and of potassium carbonate (0.65 g, 4.75 mmol) on 50 mL of acetonitrile was added and refluxed at

85 °C for 6 h. Reaction mixture was filtered and concentrated and purified by Isolera One Biotage device with a AcOEt/Hx 1:1 mobile phase and Biotage SNAP Cartridge, silica, 10 g to afford isoindoline compound **2** as grey solid (0.37 g, 85%).

^{1}H NMR (500 MHz, $CDCl_3$-*d*): δ 7.20 (s, 4H), 7.09 – 7.04 (m, 2H), 6.71 – 6.65 (m, 2H), 4.29 – 4.21 (m, 3H), 4.18 – 4.10 (m, 2H), 3.75 (dd, J = 8.7, 6.6 Hz, 1H), 3.61 (s, 3H), 3.12 – 3.03 (m, 2H).

^{13}C NMR (126 MHz, $CDCl_3$-*d*): δ 172.78, 154.50, 139.11, 130.10, 126.83, 122.36, 115.40, 77.28, 77.02, 76.77, 67.08, 55.57, 51.43, 36.56.

MS (ESI) (m/z): $[M + H]^+$ calculated for 297.1

3 Biological assay

The anticonvulsant potential of isoindoline was evaluated in adult zebrafish (*Danio rerio*) using a pentylenetetrazol (PTZ)-induced seizure model. Zebrafish were pre-treated by immersion in tanks containing different concentrations for 30 minutes before seizure induction. Diazepam at 75 μM was used as pharmacological control. Isoindoline derivate of tyrosine tested at 63 and 125 μM, while isoindolina derivate of tryptophan tested at 10 and 25 μM. Dimethyl sulfoxide al 0.1% was used as vehicle.

Following pre-treatment, zebrafish were transferred to a 10 mM PTZ solution (1 L) to induce convulsions. Behavioral responses were recorded and classified into five distinct seizure stages:

Stage 1: Time to present increased swimming activity and high frequency of opercular movement.

Stage 2: Time to present burst swimming, left and right movements, and erratic movements.

Stage 3: Time to present circling movements.

Stage 4: Time to present clonic seizure-like behavior (abnormal whole-body rhythmic muscular contraction).

Stage 5: Time to present fall to the bottom of the tank, tonic seizure-like behavior (sinking to the bottom of the tank, loss of body posture, and principally by rigid extension of the body).

The five stages are described in time in seconds [1].

4 ADMET Profiling of Selected Schiff Base Derivatives

ADMET (Absorption, Distribution, Metabolism, Excretion, and Toxicity) profiling was performed to evaluate the drug-like properties of the active compounds using ADMETLab v3.0 (https://admetlab3.scbdd.com/server/evaluationCal).[4, 5] This platform provides a comprehensive analysis of pharmacokinetic and toxicity parameters essential for drug discovery and development. Key parameters assessed include molecular weight, lipophilicity (logP), and topological polar surface area (TPSA) under physicochemical properties; human intestinal absorption (HIA), Caco-2 permeability, and bioavailability (F 50%) for absorption; plasma protein binding (PPB) and volume of distribution (VDss) for distribution; interactions with cytochrome P450 enzymes (e.g., CYP1A2 and CYP3A4) for metabolism; and plasma clearance (CL plasma) and half-life ($T_{1/2}$) for excretion. Additionally, toxicity predictions, such as hERG blockade, Ames mutagenicity, drug-induced liver injury (DILI), and carcinogenicity, were evaluated to identify potential safety concerns. The results of this profiling are summarized in Table S1, providing an overview of the key ADMET properties critical for determining the therapeutic potential of the compounds. The ADMETLab v3.0 tool was instrumental in offering a reliable, high-throughput computational framework for this systematic assessment.

Table S1: Summary of key ADMET properties for active compounds analyzed, including molecular weight, lipophilicity (logP), topological polar surface area (TPSA), absorption (Caco-2 permeability, human intestinal absorption, and bioavailability at 50%), distribution (plasma protein binding and volume of distribution), metabolism (CYP1A2 and CYP3A4 enzyme interactions), excretion (plasma clearance and half-life), and toxicity indicators (hERG blockade, Ames mutagenicity, drug-induced liver injury, and carcinogenicity).

Parameter	ETYR	ETRP	EHIS	EPHE	Diazepam	Comment
**(A) Physicochemical Property**						
Molecular Weight	297.14	320.15	271.13	281.14	284.070	Contain hydrogen atoms. Optimal: 100~600
Volume	313.127	338.733	277.079	304.337	284.526	Van der Waals volume
Density	0.949	0.945	0.979	0.924	0.998	Density = MW / Volume
nHA	4	4	5	3	3	Number of hydrogen bond acceptors. Optimal: 0~12
nHD	1	1	1	0	0	Number of hydrogen bond donors. Optimal: 0~7
nRot	5	5	5	5	1	Number of rotatable bonds. Optimal: 0~11
nRing	3	4	3	3	3	Number of rings. Optimal: 0~6

Parameter	ETYR	ETRP	EHIS	EPHE	Diazepam	Comment
MaxRing	9	9	9	9	11	Number of atoms in the biggest ring. Optimal: 0~18
nHet	4	4	5	3	4	Number of heteroatoms. Optimal: 1~15
fChar	0	0	0	0	0	Formal charge. Optimal: -4~4
nRig	17	21	16	17	19	Number of rigid bonds. Optimal: 0~30
Flexibility	0.294	0.238	0.312	0.294	0.053	Flexibility = nRot / nRig
Stereo Centers	1	1	1	1	0	Stereo Centers. Optimal: ≤ 2
TPSA	49.77	45.33	58.22	29.54	32.670	Topological Polar Surface Area. Optimal: 0~140
logS	-2.849	-3.514	-1.998	-2.882	-4.073	The logarithm of aqueous solubility value.
logP	2.333	2.923	1.111	2.905	2.823	The logarithm of the n-octanol/water distribution coefficients at pH=7.4.
logD	2.351	2.906	1.195	2.91	2.571	The logarithm of the n-octanol/water distribution coefficient.
pKa (Acid)	9.705	9.627	10.8	8.956	7.836	Acid-base dissociation constant (pKa) value represents the strength of a drug molecule's acidity or basicity.
pKa (Base)	8.029	7.317	6.275	6.547	1.11	Acid-base dissociation constant (pKa) value represents the strength of a drug molecule's acidity or basicity.
Melting Point	122.169	119.189	137.22	79.974	173.215	The predicted melting point of a compound is expressed in degrees Celsius (°C). Solid if >25°C.
Boiling Point	281.407	289.9	317.764	297.346	332.033	The predicted boiling point of a compound is expressed in degrees Celsius (°C). Gas if <25°C.
**(B) Medicinal Chemistry**						
QED	0.881	0.751	0.856	0.807	0.792	A measure of drug-likeness based on desirability; Attractive: > 0.67; Unattractive: 0.49~0.67; Too complex: < 0.34
GASA	0.0	0.0	0.0	0.0	0.0	Easy to synthesize (ES); Hard to synthesize (HS). Value indicates the probability of being difficult to synthesize.
Synth	2.0	2.0	3.0	2.0	2.0	Synthetic accessibility score. Easy if <6; Difficult if ≥6.
Fsp3	0.278	0.25	0.333	0.278	0.125	The number of sp3 hybridized carbons / total carbon count. Fsp3 ≥0.42 is suitable.
MCE-18	50.783	63.2	48.0	47.609	33.778	Medicinal Chemistry Evolution score. Suitable if ≥45.
NPscore	-0.122	-0.304	-0.369	-0.396	-0.558	Natural product-likeness score. Higher scores indicate higher natural product likelihood.
Lipinski Rule	0	0	0	0	0	MW ≤ 500; logP ≤ 5; Hacc ≤ 10; Hdon ≤ 5. One property out of range acceptable; two properties indicate poor absorption/permeability.
Pfizer Rule	0	0	0	0	0	logP > 3; TPSA < 75. Compounds with high logP and low TPSA are likely toxic.

Parameter	ETYR	ETRP	EHIS	EPHE	Diazepam	Comment
GSK Rule	0	0	0	0	0	MW ≤ 400; logP ≤ 4. Satisfying the GSK rule indicates a favorable ADMET profile.
Golden Triangle	0	0	0	0	0	200 ≤ MW ≤ 500; -2 ≤ logD ≤ 5. Indicates a favorable ADMET profile.
PAINS	0	0	0	0	0	Frequent hitters, alpha-screen artifacts, or reactive compound substructures.
ALARM NMR	1	0	0	0	0	Thiol-reactive compounds.
BMS	0	0	0	0	0	Indicates undesirable, reactive compounds.
Chelator Rule	0	0	0	0	0	Indicates chelating compounds.
Colloidal Aggregators	0.227	0.719	0.172	0.272	0.211	Category 0: non-colloidal aggregators; Category 1: colloidal aggregators.
**(C) Absorption**						
Caco-2	-4.865	-4.786	-5.365	-4.641	-4.265	Caco-2 Permeability; Optimal: higher than -5.15 Log unit
MDCK Permeability	-4.438	-4.585	-4.882	-4.522	-4.349	Low permeability: < 2 × 10^{-6} cm/s; Medium: 2-20 × 10^{-6} cm/s; High: > 20 × 10^{-6} cm/s
PAMPA	0.041	0.031	0.04	0.02	0.0	Molecules with logPeff below 2.0 classified as low-permeability; above 2.5 as high-permeability
Pgp-inhibitor	0.849	0.938	0.888	0.989	0.037	Category 0: Non-inhibitor; Category 1: Inhibitor
Pgp-substrate	0.733	0.797	0.88	0.855	0.792	Category 0: Non-substrate; Category 1: Substrate
HIA	0	0	0	0	0	Human Intestinal Absorption. Category 1: HIA+ (<30%); Category 0: HIA- (≥30%)
F 20%	0.333	0.003	0.038	0.034	0	20% Bioavailability. Category 1: F 20%+ (<20%); Category 0: F 20%- (≥20%)
F 30%	0.53	0.053	0.087	0.165	0	30% Bioavailability. Category 1: F 30%+ (<30%); Category 0: F 30%- (≥30%)
F 50%	0.954	0.383	0.263	0.533	0	50% Bioavailability. Category 1: F 50%+ (<50%); Category 0: F 50%- (≥50%)
**(D) Distribution**						
PPB	96.931	96.656	86.492	96.057	97.981	Plasma Protein Binding. Optimal: <90%. High binding indicates a low therapeutic index.
VDss	-0.031	-0.058	0.076	0.026	-0.124	Volume of Distribution. Optimal: 0.04–20 L/kg
BBB	0.023	0.037	0.033	0.114	1.0	Blood-Brain Barrier Penetration. Category 1: BBB+; Category 0: BBB-
Fu	2.791	3.794	15.123	4.204	1.772	Fraction unbound in plasma. Low: <5%; Medium: 5~20%; High: >20%
OATP1B1 inhibitor	0.996	0.988	0.942	0.968	0.0	Category 0: Non-inhibitor;
OATP1B3 inhibitor	0.99	0.983	0.883	0.954	0.0	Category 1: Inhibitor
BCRP inhibitor	0.033	0.012	0.002	0.02	0.0	
MRP1 inhibitor	0.765	0.548	0.695	0.567	0.829	
**(E) Metabolism**						
CYP1A2 inhibitor	0.97	0.992	0.038	0.96	0.987	Category 0: Non-inhibitor;
CYP1A2 substrate	0.716	0.97	0.328	0.991	1.0	Category 1: Inhibitor
CYP2C19 inhibitor	1.0	1.0	0.976	1.0	0.046	

Parameter	ETYR	ETRP	EHIS	EPHE	Diazepam	Comment
CYP2C19 substrate	0.829	0.983	0.928	0.805	1.0	Category 0: Non-substrate; Category 1: Substrate
CYP2C9 inhibitor	0.855	0.681	0.18	0.986	0.083	
CYP2C9 substrate	0.977	0.986	0.435	0.975	1.0	
CYP2D6 inhibitor	0.194	0.174	0.19	0.833	0.005	
CYP2D6 substrate	0.997	0.762	0.445	0.325	0.0	
CYP3A4 inhibitor	0.979	0.983	0.956	0.658	1.0	
CYP3A4 substrate	0.99	1.0	0.988	0.981	1.0	
CYP2B6 inhibitor	0.988	0.998	0.241	0.996	0.779	
CYP2B6 substrate	0.245	0.997	0.481	0.999	1.0	
CYP2C8 inhibitor	0.966	0.991	0.988	0.892	0.0	
HLM Stability	0.945	0.997	0.569	0.955	1.0	Human liver microsomal stability. Probability of instability: value closer to 1 indicates higher likelihood of instability.
**(F) Excretion**						
CL plasma	10.057	7.288	8.488	8.278	2.448	Plasma clearance in ml/min/kg. High clearance: >15 ml/min/kg; Moderate: 5–15 ml/min/kg; Low: <5 ml/min/kg
$T_{1/2}$	0.527	0.377	0.705	0.667	0.917	Half-life (hours). Ultra-short: <1 hour; Short: 1–4 hours; Intermediate: 4–8 hours; Long: >8 hours
**(G) Toxicity**						
hERG Blockers	0.386	0.33	0.146	0.416	0.464	Probability of being hERG+. Molecules with IC50 ≤10 μM are classified as hERG+ (Category 1).
hERG Blockers (10 μM)	0.634	0.616	0.436	0.637	0.65	Probability of being hERG+. Molecules with IC50 ≤10 μM are classified as hERG+ (Category 1).
DILI	0.086	0.294	0.63	0.14	0.175	Drug-Induced Liver Injury. Category 1: High risk; Category 0: Low risk.
AMES Mutagenicity	0.513	0.593	0.496	0.52	0.2	AMES toxicity. Category 1: Ames positive (mutagenic); Category 0: Ames negative (non-mutagenic).
Rat Oral Acute Toxicity	0.477	0.568	0.5	0.467	0.159	Probability of being toxic. Low-toxicity: >500 mg/kg; High-toxicity: <500 mg/kg.
FDAMDD	0.644	0.729	0.448	0.755	0.989	FDA Maximum Daily Dose. Category 1: Positive; Category 0: Negative.
Skin Sensitization	0.762	0.565	0.184	0.409	0.965	Probability of being a skin sensitizer. Category 1: Sensitizer; Category 0: Non-sensitizer.
Carcinogenicity	0.474	0.492	0.408	0.009	0.093	Probability of being a carcinogen. Category 1: Carcinogenic; Category 0: Non-carcinogenic.
Eye Corrosion	0.007	0.003	0.005	0.401	0.0	Probability of causing eye corrosion. Category 1: Corrosive; Category 0: Non-corrosive.
Eye Irritation	0.535	0.505	0.692	0.752	0.032	Probability of causing eye irritation. Category 1: Irritant; Category 0: Non-irritant.
Respiratory	0.761	0.812	0.676	0.996	0.23	Probability of being a respiratory toxicant. Category 1: Toxicant; Category 0: Non-toxicant.
Human Hepatotoxicity	0.63	0.654	0.653	0.669	0.646	Probability of being hepatotoxic. Category 1: Positive; Category 0: Negative.

Parameter	ETYR	ETRP	EHIS	EPHE	Diazepam	Comment
Drug-Induced Nephrotoxicity	0.335	0.552	0.506	0.511	0.812	Probability of being nephrotoxic. Category 1: Nephrotoxic; Category 0: Non-nephrotoxic.
Ototoxicity	0.43	0.524	0.506	0.415	0.254	Probability of being ototoxic. Category 1: Ototoxic; Category 0: Non-ototoxic.
Hematotoxicity	0.225	0.37	0.43	0.366	0.914	Probability of being hematotoxic. Category 1: Hematotoxic; Category 0: Non-hematotoxic.
Genotoxicity	0.874	0.785	0.976	0.706	0.999	Probability of being genotoxic. Category 1: Genotoxic; Category 0: Non-genotoxic.
RPMI-8226 Immunotoxicity	0.036	0.035	0.037	0.039	0.062	Probability of being immunotoxic. Category 1: Cytotoxic; Category 0: Non-cytotoxic.
A549 Cytotoxicity	0.08	0.07	0.026	0.058	0.014	Probability of being cytotoxic. Category 1: Cytotoxic; Category 0: Non-cytotoxic.
Hek293 Cytotoxicity	0.468	0.228	0.164	0.272	0.616	Probability of being cytotoxic. Category 1: Cytotoxic; Category 0: Non-cytotoxic.
Drug-Induced Neurotoxicity	0.748	0.816	0.89	0.826	1.0	Probability of being neurotoxic. Category 1: Neurotoxic; Category 0: Non-neurotoxic.
**(H) Environmental Toxicity**						
Bioconcentration Factors	1.348	1.571	0.829	1.509	1.374	Used for assessing risks to human health via the food chain. Unit: -$\log_{10}$[(mg/L)/(1000*MW)].
IGC_{50}	3.953	3.938	3.391	3.905	4.202	Tetrahymena pyriformis 50% growth inhibition concentration. Unit: -$\log_{10}$[(mg/L)/(1000*MW)].
LC_{50} (FM)	4.701	4.764	4.21	4.648	4.916	96-hour fathead minnow 50% lethal concentration. Unit: -$\log_{10}$[(mg/L)/(1000*MW)].
LC_{50} (DM)	5.154	5.296	4.74	5.103	5.34	48-hour Daphnia magna 50% lethal concentration. Unit: -$\log_{10}$[(mg/L)/(1000*MW)].
**(I) Tox21 Pathway**						
NR-AhR	0.038	0.833	0.071	0.016	0.014	Aryl hydrocarbon receptor. Category 1: Active; Category 0: Inactive.
NR-AR	0.002	0.002	0.004	0.001	0.022	Androgen receptor. Category 1: Active; Category 0: Inactive.
NR-AR-LBD	0.003	0.001	0.0	0.002	0.002	Androgen receptor ligand-binding domain. Category 1: Active; Category 0: Inactive.
NR-Aromatase	0.013	0.027	0.024	0.001	0.039	Aromatase. Category 1: Active; Category 0: Inactive.
NR-ER	0.071	0.126	0.05	0.157	0.261	Estrogen receptor. Category 1: Active; Category 0: Inactive.
NR-ER-LBD	0.002	0.002	0.003	0.0	0.0	Estrogen receptor ligand-binding domain. Category 1: Active; Category 0: Inactive.
NR-PPAR-gamma	0.0	0.0	0.003	0.0	0.001	Peroxisome proliferator-activated receptor gamma. Category 1: Active; Category 0: Inactive.
SR-ARE	0.02	0.012	0.0	0.005	0.237	Antioxidant response element. Category 1: Active; Category 0: Inactive.

Parameter	ETYR	ETRP	EHIS	EPHE	Diazepam	Comment
SR-ATAD5	0.0	0.001	0.007	0.0	0.986	ATPase family AAA domain-containing protein 5. Category 1: Active; Category 0: Inactive.
SR-HSE	0.003	0.018	0.023	0.001	0.001	Heat shock factor response element. Category 1: Active; Category 0: Inactive.
SR-MMP	0.008	0.004	0.006	0.001	0.02	Mitochondrial membrane potential. Category 1: Active; Category 0: Inactive.
SR-p53	0.013	0.043	0.143	0.005	0.047	Tumor suppressor protein p53. Category 1: Active; Category 0: Inactive.
**(J) Toxicophore Rules**						
Acute Toxicity Rule	0	0	0	0	0	Contains 20 substructures related to acute toxicity during oral administration.
Genotoxic Carcinogenicity Rule	0	0	0	0	1 alert	Indicates carcinogenicity or mutagenicity based on 117 substructures.
Non-Genotoxic Carcinogenicity Rule	0	0	0	0	1 alert	Indicates carcinogenicity through non-genotoxic mechanisms based on 23 substructures.
Skin Sensitization Rule	0	0	0	0	1 alert	Indicates skin irritation based on 155 substructures.
Aquatic Toxicity Rule	0	0	0	0	1 alert	Indicates toxicity to liquid (water) based on 99 substructures.
Non-Biodegradable Rule	0	0	0	0	1 alert	Indicates non-biodegradable compounds based on 19 substructures.
SureChEMBL Rule	0	0	0	0	0	Indicates MedChem unfriendly status based on 164 substructures.
FAF-Drugs4 Rule	1 alert	2 alerts	0	0	1 alert	Indicates toxic substructures (154) as per FAF-Drugs4.

5 Plots, Statistical Analysis and Visualizations

5.1 Descriptive Statistics

The statistical analysis of the data was carried out using comprehensive methods to ensure the robustness and reliability of the results. The selection of statistical tests was based on normality (Shapiro-Wilk test) and homoscedasticity (Levene's test). Depending on the assumptions met, the appropriate multiple comparison test was chosen. Statistical analysis was conducted using SPSS v29.0.2.0.

The descriptive statistics of the five stages according to the compound were based on measures of central tendency and dispersion, which are described in Table S2.

Table S2. Descriptive statistics of time to reach the five stages according to the compound

Variable	Group	Mean	Median	SEM	SD	Variance	RAIQ	Maximum	Minimum
Latency stage 1	Seizure inducer (PTZ)	31.5	29	4.94	12.09	146.3	19.3	50	15
	Vehicle (DMSO 0.1%)	29.5	27.5	2.79	6.83	46.7	12.5	40	23
	Anticonvulsant (DZP)	63.3	54	9.89	24.25	587.87	27	111	45
	ETyr (63 µM)	30.5	33.5	4.76	11.66	135.9	20	45	13
	ETyr (125 µM)	23.5	26.5	5.45	13.35	178.3	25	38	4
	ETrp (10 µM)	56.4	49	7.06	17.29	299.05	33.6	81.2	40
	ETrp (25 µM)	51.4	53.1	8.19	20.08	403.02	36.2	78	27
Latency stage 2	Seizure inducer (PTZ)	49.9	46.5	6.87	16.82	282.94	29.4	78	32.4
	Vehicle (DMSO 0.1%)	48	47.1		9.51		13.5	65	38
	Anticonvulsant (DZP)	141.6	137	16.43	40.25	1620.21	74.7	187.2	83.4
	ETyr (63 µM)	57.7	62	8.02	19.63	385.52	28.4	74	21
	ETyr (125 µM)	42.9	47	7.86	19.24	370.19	38.4	66.6	18
	ETrp (10 µM)	163.4	162.5	13.59	33.28	1107.42	68	200	121
	ETrp (25 µM)	243.4	239.2	31.73	77.72	6040.73	99.4	385.2	158.4
Latency stage 3	Seizure inducer (PTZ)	74	75.6	13.79	33.79	1142.18	59.7	121.8	30
	Vehicle (DMSO 0.1%)	91.9	89	4.00	9.81	25579.61	18.3	107.4	82.2
	Anticonvulsant (DZP)	192.9	186	23.79	58.28	3396.09	82.5	300	136.2
	ETyr (63 µM)	136.9	133.4	34.27	83.94	7046.57	150.1	249.6	32
	ETyr (125 µM)	79.7	59.1	27.56	67.5	4556.36	62.7	213.6	24
	ETrp (10 µM)	311	296.9	30.57	74.91	5610.69	106.3	443.8	228.8
	ETrp (25 µM)	410	376.2	65.29	159.94	25579.61	270.4	668.4	246.8
Latency stage 4	Seizure inducer (PTZ)	242.1	250.5	24.69	60.48	3657.35	77	329.4	143.8
	Vehicle (DMSO 0.1%)	276.9	280.3	8.43	20.65	426.59	29.1	300	240

	Anticonvulsant (DZP)	892.5	892	40.33	98.79	9758.87	124.2	1047.6	747
	ETyr (63 μM)	272.1	205.5	85.02	208.27	43374.84	433.6	559	78.6
	ETyr (125 μM)	161.4	146.1	33.9	83.07	6900.19	173.9	267.6	67.2
	ETrp (10 μM)	840.2	832.4	32.28	79.06	6250.89	88.8	977	732.8
	ETrp (25 μM)	726.9	739.2	85.43	209.25	43784.9	402	991.2	439.4
Latency stage 5	Seizure inducer (PTZ)	324.3	362.5	30.56	74.86	5604.17	126.8	384	199
	Vehicle (DMSO 0.1%)	304.2	301.5	6.13	15.01	225.29	23.3	330.6	288.6
	Anticonvulsant (DZP)	1006.7	960	46.47	113.84	12958.73	170.3	1217.4	923.4
	ETyr (63 μM)	356.6	226.5	138.69	339.71	115402.72	522	976	81
	ETyr (125 μM)	247.1	219.9	69.33	169.83	28841.13	227.5	560.4	85.2
	ETrp (10 μM)	854.9	852.2	32.05	78.49	6161.14	76.6	986	739.4
	ETrp (25 μM)	797	117.3	62.79	153.82	23660.27	246.6	1025	580.6
Abbreviations: SEM: Standard error of the mean, SD standard deviation, RAIQ: interquartile range.									

Latency stage 1

A one-way ANOVA was performed to assess the effects of different compounds on the PTZ-induced seizure model in zebrafish (*Danio rerio*).

Normality and Homoscedasticity Testing

(1) Shapiro-Wilk test was used to check for normality.
(2) Levene's test assessed homoscedasticity (equal variance) among groups.
(3) Even though some groups did not meet normality, the assumption of homoscedasticity was met, making ANOVA robust to these violations.

Table S3. Compliance with the assumptions of normality and homoscedasticity of stage 1, and result of one-way ANOVA

Variable	Group	Shapiro-Wilk	Levene	ANOVA
Latency stage 1	Seizure inducer (PTZ)	0.88	0.18	<0.001[a]**
	Vehicle (DMSO 0.1%)	0.35		
	Anticonvulsant (DZP)	0.02*		
	ETyr (63 μM)	0.8		
	ETyr (125 μM)	0.59		
	ETrp (10 μM)	0.12		
	ETrp (25 μM)	0.59		

*Group that does not meet the assumption of normality by the Shapiro-Wilks test.
**Statistically significant *p-value* in the ANOVA test
[a]One-Way ANOVA

**Table S4.** Detailed statistical results for the one-way ANOVA test applied to the stage 1						
**Variable**	**Comparison**	**Sum of squares**	**Degrees of freedom**	**Quadratic mean**	**F**	***P-value***
**Latency stage 1**	Between groups	8891.739	6	1481.957	5.772	**<0.001***
	Within groups	985.687	35	256.734		
	Total	17877.426	41			
*Statistically significant *p-value* in the ANOVA test						

Post-Hoc Analysis

To determine significant pairwise differences, a Dunnett post-hoc test was conducted. This test controls the family-wise error rate, ensuring that multiple comparisons maintain statistical reliability, and is extremely useful when comparing a control group.

Statistical Significance

A p-value < 0.05 was considered statistically significant.

**Table S5**. Dunnett post-hoc test: Pairwise p-values for stage 1 in the one-way ANOVA.							
	Seizure inducer (PTZ)	Vehicle (DMSO 0.1%)	Anticonvulsant (DZP)	ETyr (63 μM)	ETyr (125 μM)	ETrp (10 μM)	ETrp (25 μM)
Seizure inducer (PTZ)	NA	0.90	**0.004***	0.88	0.98	**0.02***	0.08
*Statistically significant *p-value* in Dunnett post hoc test							

Latency stage 2

A one-way ANOVA was performed to assess the effects of different compounds on the PTZ-induced seizure model in zebrafish (*Danio rerio*).

Normality and Homoscedasticity Testing

(1) Shapiro-Wilk test was used to check for normality.
(2) Levene's test assessed homoscedasticity (equal variance) among groups.
(3) Even though some groups did not meet normality, the assumption of homoscedasticity was met, making ANOVA robust to these violations

Table S6. Compliance with the assumptions of normality and homoscedasticity of stage 2, and result of one-way ANOVA				
**Variable**	**Group**	**Shapiro-Wilk**	**Levene**	**ANOVA**
Latency stage 2	Pro-Seizure inducer (PTZ)	0.53	0.07	<0.001[a]*
	Vehicle (DMSO 0.1%)	0.42		
	Anticonvulsant (DZP)	0.60		
	ETyr (63 μM)	0.10		
	ETyr (125 μM)	0.45		
	ETrp (10 μM)	0.43		
	ETrp (25 μM)	0.25		

*Group that does not meet the assumption of normality by the Shapiro-Wilks test.
**Statistically significant *p-value* in the ANOVA test
[a]One-Way ANOVA

Table S7. Detailed statistical results for the one-way ANOVA test applied to the stage 1

Variable	Comparison	Sum of squares	Degrees of freedom	Quadratic mean	F	*P-value*
**Latency stage 2**	Between groups	217587.730	6	36264.622	25.648	**<0.001***
	Within groups	49487.387	35	1413.925		
	Total	267075.116	41			

*Statistically significant *p-value* in the ANOVA test

Post-Hoc Analysis

To determine significant pairwise differences, a Dunnett post hoc test was conducted. This test controls the family-wise error rate, ensuring that multiple comparisons maintain statistical reliability, and is extremely useful when comparing a control group.

Statistical Significance

A *p-value* < 0.05 was considered statistically significant.

Table S8. Dunnett post-hoc test: Pairwise p-values for stage 2 in the one-way ANOVA

	Seizure inducer (PTZ)	Vehicle (DMSO 0.1%)	Anticonvulsant (DZP)	ETyr (63 μM)	ETyr (125 μM)	ETrp (10 μM)	ETrp (25 μM)
Seizure inducer (PTZ)	NA	0.88	**<0.001***	0.74	0.29	**<0.001***	**<0.001***

*Statistically significant *p-value* in Dunnett post hoc test

Latency stage 3

A Kruskal-Wallis test was performed to assess the effects of different compounds on the PTZ-induced seizure model in zebrafish (*Danio rerio*).

Normality and Homoscedasticity Testing

(1) Shapiro-Wilk test assessed normality of data distribution.
(2) Levene's test determined homoscedasticity (equal variance) across groups.
(3) Based on these results:
Kruskal-Wallis test was applied.

Table S9. Compliance with the assumptions of normality and homoscedasticity of stage 3, and result of Kruskal-Wallis test

Variable	Group	Shapiro-Wilk	Levene	Kruskal-Wallis test (*p-value*)
Latency stage 3 (s)	Pro-Seizure inducer (PTZ)	0.94	0.001**	<0.001***
	Vehicle (DMSO 0.1%)	0.41		
	Anticonvulsant (DZP)	0.20		
	ETyr (63 µM)	0.74		
	ETyr (125 µM)	0.01*		
	ETrp (10 µM)	0.52		
	ETrp (25 µM)	0.53		

*Group that does not meet the assumption of normality by the Shapiro-Wilks test.
**Group that does not meet the assumption of homoscedasticity by the Levene test.
***Statistically significant *p-value* in the Kruskal-Wallis test

Post-Hoc Analysis

For these non-parametric datasets, Mann-Whitney U test was conducted to compare pairs of treatment groups.

Bonferroni correction was applied to account for multiple comparisons, using the formula:

$$p' = 1 - (1 - p)^c$$

Where c is the number of comparisons.

- Significant p-values ($p < 0.05$) were adjusted using this Bonferroni method.
- Values between identical groups (e.g., Control vs. Control) were marked as "Not Applicable", as they do not meet independence assumptions.
- To avoid redundancy, pairwise p-values were reported only once.

**Table S10**. Mann-Whitney U test results: Pairwise p-values for stage 3, with Bonferroni correction for Kruskal-Wallis post hoc analysis.							
	Seizure inducer (PTZ)	Vehicle (DMSO 0.1%)	Anticonvulsant (DZP)	ETyr (63 μM)	ETyr (125 μM)	ETrp (10 μM)	ETrp (25 μM)
Seizure inducer (PTZ)	NA	0.25	**<0.01[a]***	0.25	0.25	**<0.01[a]***	**<0.01[a]***
Vehicle (DMSO 0.1%)	-	NA	**<0.01[a]***	0.25	0.14[a]	**<0.01[a]***	**<0.01[a]***
Anticonvulsant (DZP)	-	-	NA	0.25	0.14[a]	0.14[a]	0.14[a]
ETyr (63 μM)	-	-	-	NA	0.14[a]	**<0.01[a]***	**<0.01[a]***
ETyr (125 μM)	-	-	-	-	NA	**<0.01[a]***	**<0.01[a]***
ETrp (10 μM)	-	-	-	-	-	NA	0.25
ETrp (25 μM)	-	-	-	-	-	-	NA
[a]*p-value* obtained from the Mann-Whitney U test, corrected by Bonferroni method<br>*Statistically significant *p-value*							

Latency stage 4

A Kruskal-Wallis test was performed to assess the effects of different compounds on the PTZ-induced seizure model in zebrafish (*Danio rerio*).

Normality and Homoscedasticity Testing

(1) Shapiro-Wilk test assessed normality of data distribution.
(2) Levene's test determined homoscedasticity (equal variance) across groups.
(3) Based on these results:
    a) Kruskal-Wallis test was applied.

**Table S11. Compliance with the assumptions of normality and homoscedasticity of stage 4, and result of Kruskal-Wallis test**				
**Variable**	**Group**	**Shapiro-Wilk**	**Levene**	**Kruskal-Wallis test (*p-value*)**
Latency stage 4 (s)	Pro-Seizure inducer (PTZ)	0.68	<0.001**	<0.001***
	Vehicle (DMSO 0.1%)	0.49		
	Anticonvulsant (DZP)	0.81		
	ETyr (63 μM)	0.14		
	ETyr (125 μM)	0.39		
	ETrp (10 μM)	0.44		
	ETrp (25 μM)	0.92		
*Group that does not meet the assumption of normality by the Shapiro-Wilks test.<br>**Group that does not meet the assumption of homoscedasticity by the Levene test.<br>***Statistically significant *p-value* in the Kruskal-Wallis test				

Post-Hoc Analysis

For these non-parametric datasets, Mann-Whitney U test was conducted to compare pairs of treatment groups.

Bonferroni correction was applied to account for multiple comparisons, using the formula:

$$p' = 1 - (1 - p)^c$$

Where c is the number of comparisons.

- Significant p-values ($p < 0.05$) were adjusted using this Bonferroni method.
- Values between identical groups (e.g., Control vs. Control) were marked as "Not Applicable", as they do not meet independence assumptions.
- To avoid redundancy, pairwise *p-values* were reported only once.

Table S12. Mann-Whitney U test results: Pairwise p-values for stage 3, with Bonferroni correction for Kruskal-Wallis post-hoc analysis.

	Seizure inducer (PTZ)	Vehicle (DMSO 0.1%)	Anticonvulsant (DZP)	ETyr (63 µM)	ETyr (125 µM)	ETrp (10 µM)	ETrp (25 µM)
Seizure inducer (PTZ)	NA	0.14[a]	**<0.01[a]***	0.25	0.25	**<0.01[a]***	**<0.01[a]***
Vehicle (DMSO 0.1%)	-	NA	**<0.01[a]***	0.25	0.14[a]	**<0.01[a]***	**<0.01[a]***
Anticonvulsant (DZP)	-	-	NA	**<0.01[a]***	**<0.01[a]***	0.14[a]	0.25
ETyr (63 µM)	-	-	-	NA	0.25	**<0.01[a]***	0.14[a]
ETyr (125 µM)	-	-	-	-	NA	**<0.01[a]***	**<0.01[a]***
ETrp (10 µM)	-	-	-	-	-	NA	0.25
ETrp (25 µM)	-	-	-	-	-	-	NA

[a]p-value obtained from the Mann-Whitney U test, corrected by Bonferroni method
*Statistically significant *p-value*

Latency stage 5

A Kruskal-Wallis test was performed to assess the effects of different compounds on the PTZ-induced seizure model in zebrafish (*Danio rerio*).

Normality and Homoscedasticity Testing

(1) Shapiro-Wilk test assessed normality of data distribution.
(2) Levene's test determined homoscedasticity (equal variance) across groups.
(3) Based on these results:

Kruskal-Wallis test was applied.

**Table S13. Compliance with the assumptions of normality and homoscedasticity of stage 3, and result of Kruskal-Wallis test**				
**Variable**	**Group**	**Shapiro-Wilk**	**Levene**	**Kruskal-Wallis test (*p-value*)**
Latency stage 5 (s)	Pro-Seizure inducer (PTZ)	0.06	0.003**	<0.001***
	Vehicle (DMSO 0.1%)	0.52		
	Anticonvulsant (DZP)	0.05		
	ETyr (63 µM)	0.10		
	ETyr (125 µM)	0.21		
	ETrp (10 µM)	0.27		
	ETrp (25 µM)	0.95		
*Group that does not meet the assumption of normality by the Shapiro-Wilks test.<br>**Group that does not meet the assumption of homoscedasticity by the Levene test.<br>***Statistically significant *p-value* in the Kruskal-Wallis test				

Post-Hoc Analysis

For these non-parametric datasets, Mann-Whitney U test was conducted to compare pairs of treatment groups.

Bonferroni correction was applied to account for multiple comparisons, using the formula:

$$p' = 1 - (1 - p)^c$$

Where c is the number of comparisons.

- Significant p-values ($p < 0.05$) were adjusted using this Bonferroni method.
- Values between identical groups (e.g., Control vs. Control) were marked as "Not Applicable", as they do not meet independence assumptions.
- To avoid redundancy, pairwise p-values were reported only once.

**Table S14**. Mann-Whitney U test results: Pairwise p-values for stage 3, with Bonferroni correction for Kruskal-Wallis post-hoc analysis.							
	Seizure inducer (PTZ)	Vehicle (DMSO 0.1%)	Anticonvulsant (DZP)	ETyr (63 µM)	ETyr (125 µM)	ETrp (10 µM)	ETrp (25 µM)
Seizure inducer (PTZ)	NA	0.25	**<0.01[a]***	0.25	0.25	0.25	**<0.01[a]***
Vehicle (DMSO 0.1%)	-	NA	**<0.01[a]***	0.25	0.14[a]	**<0.01[a]***	**<0.01[a]***
Anticonvulsant (DZP)	-	-	NA	0.14[a]	**<0.01[a]***	0.14[a]	0.14[a]
ETyr (63 µM)	-	-	-	NA	0.99	0.14[a]	0.14[a]
ETyr (125 µM)	-	-	-	-	NA	**<0.01[a]***	**<0.01[a]***
ETrp (10 µM)	-	-	-	-	-	NA	0.25
ETrp (25 µM)	-	-	-	-	-	-	NA
[a]*p-value* obtained from the Mann-Whitney U test, corrected by Bonferroni method<br>*Statistically significant *p-value*							

6 Structure-Activity Relationship (SAR)

6.1 Aromatic Preference and Workflow

6.1.1 Library Design: Sixteen Isoindoline Esters Derived from Natural Amino Acids

To systematically explore the structural determinants of $GABA_A$ receptor modulation via π–π interactions, a focused library of sixteen 2-(isoindolin-2-yl) esters was constructed. Each compound in this series was generated by esterification of the isoindoline core with the methyl ester of a natural L-α-amino acid, producing a series of analogs that varied exclusively at the side chain (R-group). This approach maintained a consistent molecular scaffold, enabling reliable comparison of physicochemical properties and binding behaviors across structurally diverse side chains.

The sixteen amino acids selected for this series span the major chemical categories of natural residues:

Aromatic: Tryptophan, Tyrosine, Phenylalanine, Histidine

Aliphatic (non-polar): Alanine, Valine, Leucine, Isoleucine, Methionine

Polar (uncharged): Serine, Threonine, Cysteine, Asparagine, Glutamine

Polar (charged): Glutamic acid, Arginine

Special case/backbone variation: Glycine, β-Alanine, 4-Aminobutyric acid

This diversity allows the evaluation of side chain effects on binding affinity, ADMET profiles, and ultimately, CNS-targeting potential within a consistent molecular context.

6.1.2 Grouping Compounds by Side Chain Type

To assess the impact of side chain chemistry on receptor interaction, compounds were grouped as follows:

Group A: Aromatic Residues (n = 4)

ETrp (Tryptophan); ETyr (Tyrosine), EPhe (Phenylalanine), EHis (Histidine)

Group B: Aliphatic Residues (n = 6)

EAla (Alanine); EVal (Valine); ELeu (Leucine); EIso (Isoleucine); EMet (Methionine); E4-Ab (4-Aminobutyric acid)

Group C: Polar Residues (n = 5)

ESer (Serine); EThr (Threonine); ECys (Cysteine); EGlu (Glutamate); Eβ-Ala (β-Alanine)

Group D: Special Case (n = 1)

EGly (Glycine) — lacks a true side chain; serves as a baseline for flexibility effects

The aromatic group (Group A) was hypothesized to have superior interaction potential at the benzodiazepine site due to π–electron delocalization, enabling stacking interactions with aromatic residues on the receptor.

6.1.3 Summary of Core Properties

The results of the physicochemical analysis and docking evaluations are summarized in Table S15, which includes molecular weight, logP, TPSA, H-bonding potential, and docking energy for each compound. This data supports initial filtering and illustrates the clustering of top performers (Trp, Tyr, His, Phe) around CNS-compatible parameters.

Table S15. Physicochemical descriptors (Lipinski-relevant) and Molecular docking of 2-(isoindolin-2-yl) esters derived from natural amino acids.

Compound	Amino Acid	Aromatic	MW (Da)	LogP	TPSA (Å²)	HBD	HBA	Binding Energy (kcal/mol)	ΔG_{bind} (kcal/mol)
DZP	Diazepam	Yes	284.74	2.99	32.67	2	0	–9.3	–77.87
ETrp (2)	Tryptophan	Yes	320.39	2.78	45.33	3	1	–9.8	–168.05
ETyr (14)	Tyrosine	Yes	297.35	2.49	49.77	4	1	–10.0	–60.97
EPhe (3)	Phenylalanine	Yes	281.35	2.78	29.54	3	0	–9.0	–
EHis (1)	Histidine	Yes	271.31	1.02	58.22	4	1	–9.3	–
EAla (6)	Alanine	No	205.25	1.56	29.54	3	0	-8	–
EVal (10)	Valine	No	233.31	2.2	29.54	3	0	-7.8	–
ELeu (12)	Leucine	No	247.33	2.59	29.54	3	0	-8	–
EIso (13)	Isoleucine	No	247.33	2.59	29.54	3	0	-8.3	–
EMet (11)	Methionine	No	265.37	2.29	54.84	3	0	-8.2	–
E4-Ab (7)	4-Aminobutyric	No	219.28	1.95	29.54	3	0	-7.5	–
ESer (5)	Serine	No	221.25	0.53	49.77	4	1	-8.1	–
EThr (9)	Threonine	No	235.28	0.92	49.77	4	1	-8	–
ECys (15)	Cysteine	No	237.32	1.47	68.34	3	0	-7.6	–
EGlu (16)	Glutamate	No	277.32	1.49	55.84	5	0	-8.6	–
EGly (4)	Glycine	No	191.23	1.17	29.54	3	0	-7.2	–
Eβ-Ala (8)	β-Alanine	No	205.25	1.56	29.54	3	0	-7.7	–

6.2 Physicochemical and ADMET Filtering

6.2.1 Physicochemical Space and CNS Compatibility

An initial analysis of molecular weight (MW), topological polar surface area (TPSA), and logP values was used to assess drug-likeness and blood–brain barrier (BBB) permeability of the sixteen isoindoline esters. Key thresholds relevant to CNS drug candidates were applied:

1) MW < 500 Da (Lipinski rule)
2) logP between 2.0 and 3.5 for optimal CNS penetration
3) TPSA < 60 Å² is preferred for BBB permeability

Aromatic compounds such as ETrp, ETyr, and EPhe fell within this ideal window, whereas polar residues like ECys, ESer, EGlu, and EThr showed elevated TPSA values (>60 Å²), making them unlikely BBB-penetrant candidates.

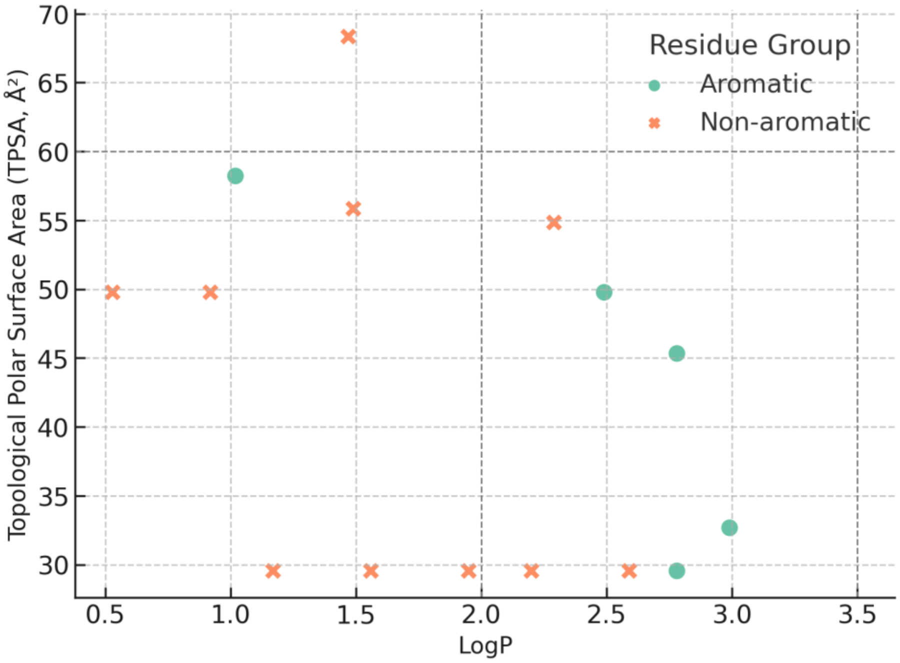

Figure S1: Distribution of LogP and TPSA values for 2-(isoindolin-2-yl) esters, categorized by side chain type. Aromatic residues cluster within the CNS-permeable window (LogP 2.0–3.5, TPSA < 60 Å²), while non-aromatic derivatives are more scattered, often outside optimal CNS drug-like space.

6.2.2 Hydrogen Bonding Profiles

Aromatic derivatives (Trp, Tyr, His) offered an optimal number of hydrogen bond donors and acceptors (HBD: 3–4; HBA: 1), supporting both BBB permeability and protein binding potential. Polar compounds exceeded these numbers, introducing desolvation penalties and reducing receptor affinity.

6.2.3 ADMET Prediction Results

Using ADMETlab 2.0 and SwissADME, additional pharmacokinetic parameters were assessed:

1) All aromatic esters showed favorable BBB permeability scores.
2) Aliphatic and charged derivatives (e.g., EAsp, EGlu, EArg) failed one or more ADMET criteria.
3) Trp and Tyr derivatives showed moderate to high GI absorption, low toxicity scores, and no structural alerts in the Ames test.

6.2.4 Conclusion of Filtering Step

Only aromatic amino acid-derived esters passed all three computational stages (physicochemical filters, ADMET criteria, and docking energy cutoffs), justifying their prioritization for molecular dynamics and in vivo testing. A correlation plot (Figure S1) shows the clustering of these candidates within the CNS-compatible physicochemical space.

6.3 Molecular Docking & π–π Interaction Profile

6.3.1 Docking Score Comparison and Prioritization

All sixteen isoindoline esters were docked to the benzodiazepine binding site of the GABAA receptor (PDB ID: 6D6U) using AutoDock Vina. The binding site was defined at the α1–γ2 interface, where classical ligands such as diazepam bind. The docking scores for all compounds were ranked and compared.

Aromatic derivatives consistently achieved higher (more negative) docking scores, with ETyr (–10.0 kcal/mol) and ETrp (–9.8 kcal/mol) outperforming DZP (–9.3 kcal/mol). EPhe and EHis also scored in the favorable range (–9.0 and –9.3 kcal/mol, respectively). In contrast, non-aromatic compounds scored below –8.0, with most ranging between –6.1 and –7.5 kcal/mol.

6.3.2 Interaction Mapping and Pose Analysis

Interaction analysis revealed that high-scoring aromatic esters engaged in multiple π–π stacking interactions with aromatic residues in the binding pocket:

ETrp: parallel displaced and edge-to-face interactions with Phe77, Tyr159, and Tyr210

ETyr: π–π stacking with Tyr159 and Phe77, and hydrogen bonding via the phenolic OH

EPhe: single π-stacking interaction with Phe77; lacked additional hydrogen bonding

EHis: weak π-interaction via imidazole ring; less planar and less electron-rich

These interactions aligned with those observed for diazepam and validate the structural mimicry of the synthetic analogs.

6.3.3 Receptor Environment and Aromatic Selectivity

The benzodiazepine binding site is characterized by a hydrophobic cleft flanked by aromatic residues. The π-stacking capacity of the ligand is essential for binding stability in this microenvironment. Aromatic amino acids provided the necessary π-surface area and rigidity to complement the binding site geometry, while aliphatic and polar derivatives failed to anchor via π–π interactions.

6.3.4 Conclusion

The docking studies clearly indicate that aromatic amino acid-derived isoindoline esters possess superior binding potential at the $GABA_A$ receptor, consistent with the receptor's aromatic microenvironment. This validates their prioritization and supports further investigation of π-extended or heteroaromatic analogs for enhanced receptor engagement. Figures S2 and S3 provide pose overlays and interaction maps highlighting key π–π stacking interactions.

6.4 Binding Energy Landscape (Metadynamics)

6.4.1 Objective and Rationale

To complement the docking results and evaluate the dynamic stability of receptor–ligand interactions, metadynamics simulations were performed for selected compounds: ETrp, ETyr, and diazepam (DZP). These simulations were designed to probe the free energy surface (FES) associated with ligand binding at the benzodiazepine site of the $GABA_A$ receptor and estimate the binding free energy (ΔG_{bind}) in a more realistic, solvent-accessible dynamic context.

6.4.2 Simulation Protocol

1) Metadynamics simulations were conducted using the PLUMED plugin in conjunction with GROMACS.
2) The simulations were run for 60 ns with collective variables (CVs) defined as the center-of-mass distance between the ligand and binding site residues (Phe77, Tyr159, Tyr210).
3) Gaussian hills were deposited every 500 steps to accelerate sampling.

6.4.3 Free Energy Profiles

The reconstructed free energy landscapes for each compound revealed distinct minima corresponding to energetically favorable bound states:

1) **ETrp** exhibited a deep and well-defined minimum (ΔG_{bind} = –168.05 kcal/mol), suggesting a highly stable binding mode with persistent π–π interactions and minimal conformational drift.
2) **ETyr** showed a moderately deep minimum (ΔG_{bind} = –60.97 kcal/mol), indicating transient but significant binding stability, likely aided by its dual π-stacking and hydrogen bonding.
3) **DZP** displayed a ΔGbind of –77.87 kcal/mol, consistent with its known efficacy as a $GABA_A$ modulator and serving as a reference benchmark.

6.4.4 Interpretation of Binding Modes

1) The deep energy well observed for ETrp correlates with its extended aromatic surface (indole ring), capable of maintaining multipoint π-stacking throughout the simulation.
2) The shallower energy minimum for ETyr may reflect flexibility in the hydroxyl group orientation and dynamic fluctuations in H-bonding.
3) The comparison underscores that aromaticity alone is not sufficient; stacking geometry, electron density, and ring orientation play vital roles in dictating binding persistence.

6.4.5 Summary

These metadynamics results corroborate the docking analysis and further validate ETrp as the most promising candidate based on binding free energy, interaction stability, and receptor complementarity. Figure S1 shows the full free energy surfaces, highlighting the relative depth and shape of the binding wells.

These dynamic simulations provide a robust energetic rationale for prioritizing Trp and Tyr derivatives in experimental assays and future structural optimization.

6.5 Receptor-Specific Interpretation

6.5.1 $GABA_A$ Receptor Binding Site Characteristics

The benzodiazepine binding site of the $GABA_A$ receptor is located at the interface between the α1 and γ2 subunits and is lined with aromatic and hydrophobic residues, including Phe77, Tyr159, His102, and Tyr210. Structural studies (e.g., cryo-EM, PDB ID: 6D6U) have shown that ligands like diazepam engage in strong π–π stacking and van der Waals interactions at this site.

6.5.2 Implications for Ligand Design

Given the aromatic density of the binding site, ligands capable of forming π–π interactions are strongly favored. Our docking and metadynamics data confirm that aromatic amino acid esters, especially ETrp and ETyr possess the correct shape, electronic features, and rigidity to align with these residues and form stabilizing non-covalent interactions.
Non-aromatic or highly polar ligands are at a disadvantage due to:

1) Lack of π-electron systems needed for stacking
2) Increased desolvation penalties due to excessive polarity (TPSA > 60 $Å^2$)
3) Flexible or extended side chains that reduce conformational complementarity

6.5.3 Mimicry of Classical Benzodiazepines

ETrp and ETyr share key features with diazepam:

1) Aromaticity enabling stacking
2) Appropriate logP (~2.5–3.0)
3) Compact, semi-rigid structure

These results reinforce the importance of molecular mimicry in rational design and support the hypothesis that 2-(isoindolin-2-yl) esters with aromatic side chains can act as functional analogs of classical benzodiazepines.

6.5.4 Conclusion

The receptor-specific binding environment favors π-rich ligands, explaining the consistent computational superiority of aromatic esters. These insights justify their prioritization in synthesis campaigns and serve as a foundation for designing next-generation analogs with improved potency and selectivity.

6.6 Comparative SAR Insights and Outliers

6.6.1 Ranking and Performance Clustering

A summary of docking scores and metadynamics results revealed a clear separation between aromatic and non-aromatic compounds in terms of receptor affinity and predicted stability. ETrp and ETyr ranked at the top across all metrics, followed by EPhe and EHis with slightly reduced performance. Aliphatic and polar derivatives clustered at the bottom with docking scores above –8.0 kcal/mol and no detectable π-stacking.

6.6.2 Outlier Analysis

Among the aromatic derivatives, **EPhe** and **EHis** performed moderately well in docking but were not selected for in vivo testing. This was due to:

1) **EPhe**: Lacked polar functional groups, offering only mono-site π-stacking; no H-bonding potential.
2) **EHis**: Imidazole ring is less electron-rich and partially protonated at physiological pH, reducing π-stacking ability.

Their physicochemical profiles (logP and TPSA) were acceptable, but not optimal compared to ETyr and ETrp. The latter two showed superior ADMET profiles, dual interaction capacity (π–π and H-bond), and broader receptor engagement.

6.6.3 Non-Aromatic Class Performance

All non-aromatic esters consistently underperformed across docking and ADMET filters. The absence of aromatic moieties precluded π–π stacking, and many polar residues exceeded TPSA or HBD/HBA thresholds. This class included:

1) High-polarity amino acids: Glu, Ser, Thr, Cys → high TPSA, failed BBB
2) Small/flexible residues: Gly, β-Ala, 4-Ab → insufficient contact points
3) Aliphatic side chains: Ala, Val, Leu → weak van der Waals, no aromatic fit

6.6.4 SAR Summary Table Reference

The comparative data is compiled in Table S15 and visualized in Figure S1. These clearly support the selective advantage of electron-rich, spatially complementary aromatic systems for $GABA_A$ receptor engagement.

6.7 Design Implications and Next-Generation Analogs

6.7.1 Key Design Principles

The following molecular features emerged as critical for potent GABAA receptor modulation:

1) **Extended π-surface**: Enables multipoint stacking with aromatic residues
2) **Moderate logP (2.5–3.0)**: Ensures CNS penetration while avoiding lipophilic toxicity
3) **TPSA < 60 Å²**: Maintains BBB permeability
4) **H-bonding functionality**: Provides auxiliary stabilization (e.g., Tyr OH)
5) **Conformational rigidity**: Minimizes entropic penalty upon binding

6.7.2 Recommended Modifications

Based on these principles, future analogs may benefit from:

1) **π-Extended groups**: e.g., naphthyl, biphenyl, or fluorene instead of phenyl
2) **Heteroaromatics**: e.g., thiophene, indazole to modulate electronic properties

3) **Substituent tuning**: e.g., 4-F, 4-OCH_3 on phenyl to influence stacking energy and polarity
4) **Non-natural amino acids**: e.g., phenylglycine, β-naphthylalanine to explore steric and electronic diversity

6.7.3 Broader Application

These findings can inform the design of other CNS-active scaffolds targeting aromatic-rich binding sites, including serotonergic, dopaminergic, or opioid receptors, where similar stacking mechanisms apply.

6.7.4 Conclusion

This SAR investigation demonstrates the strong predictive power of integrating physicochemical filtering, ADMET profiling, docking, and metadynamics simulations. The insights offer a clear path toward rational design of optimized ligands with enhanced selectivity and pharmacokinetic balance.

6.8 SAR Conclusion

This comprehensive SAR analysis of sixteen 2-(isoindolin-2-yl) esters underscores the central role of aromaticity, π–π stacking potential, and physicochemical balance in determining $GABA_A$ receptor binding affinity and CNS relevance. Aromatic amino acid-derived compounds, particularly ETrp and ETyr, consistently outperformed their aliphatic and polar counterparts across all computational stages: physicochemical screening, ADMET filtering, molecular docking, and metadynamics simulations.
The structural features of the $GABA_A$ receptor benzodiazepine site inherently favor π-rich ligands with spatial complementarity, moderate polarity, and rigid conformations. The ability of Trp and Tyr derivatives to establish multipoint interactions through both π–π stacking and hydrogen bonding contributes to their superior predicted binding energies and pharmacokinetic profiles. Conversely, non-aromatic compounds suffered from poor binding

geometry, limited contact surface area, and ADMET liabilities such as high TPSA or insufficient lipophilicity.

By integrating multiple computational layers into a coherent evaluation framework, this SAR analysis provides not only mechanistic insights into binding preferences but also clear directives for future analog development. The findings support the prioritization of aromatic scaffolds, especially those incorporating π-extended or heteroaromatic systems and establish a rational foundation for next-generation CNS ligand design targeting the $GABA_A$ receptor. Collectively, this SAR study validates the hypothesis that π-interaction-driven design, supported by dynamic and energetic modeling, is an effective strategy for developing selective, brain-penetrant GABAergic agents.

7 NMR and MS Specters

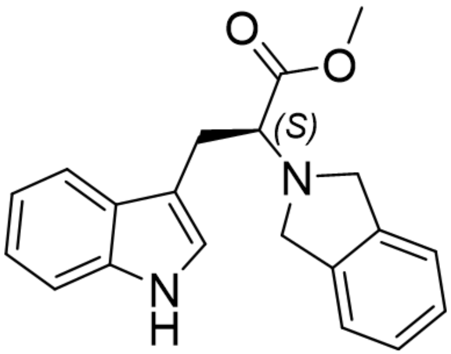

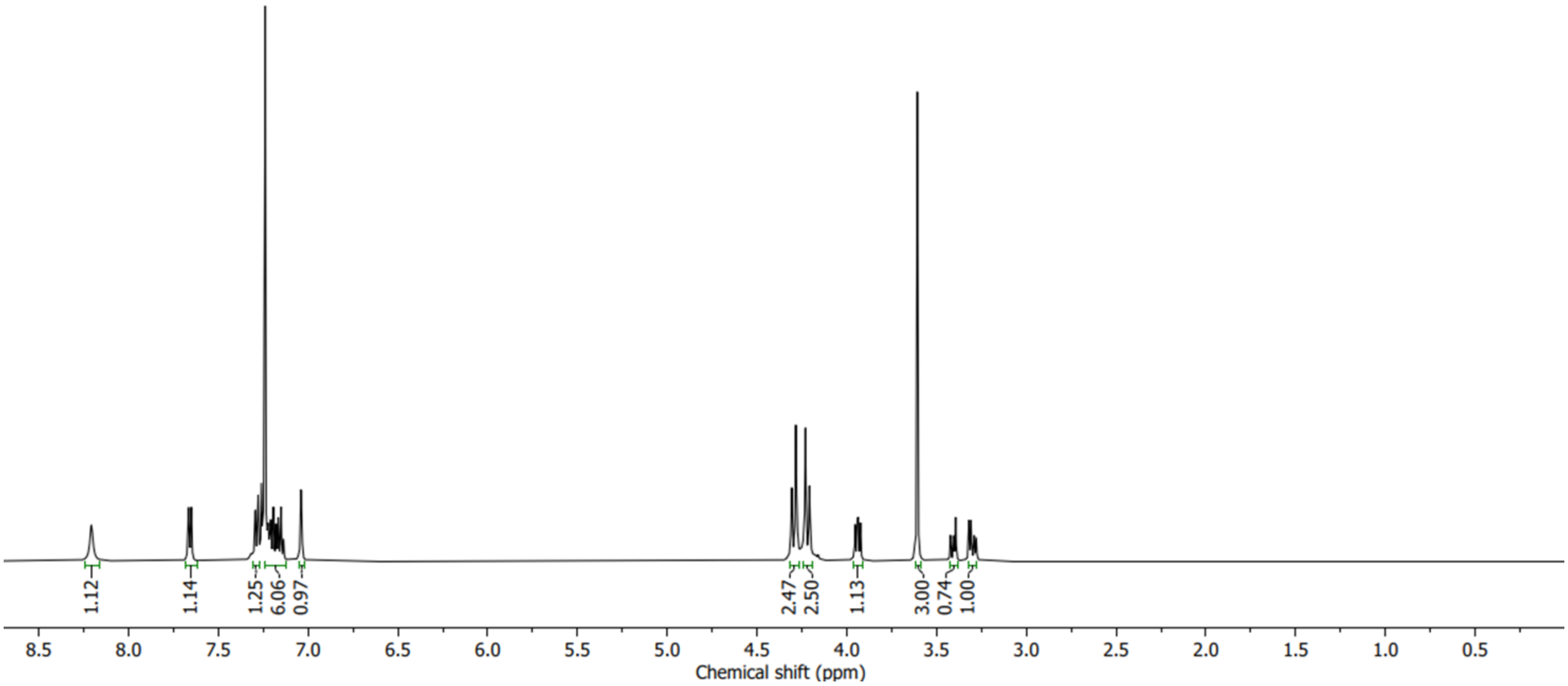

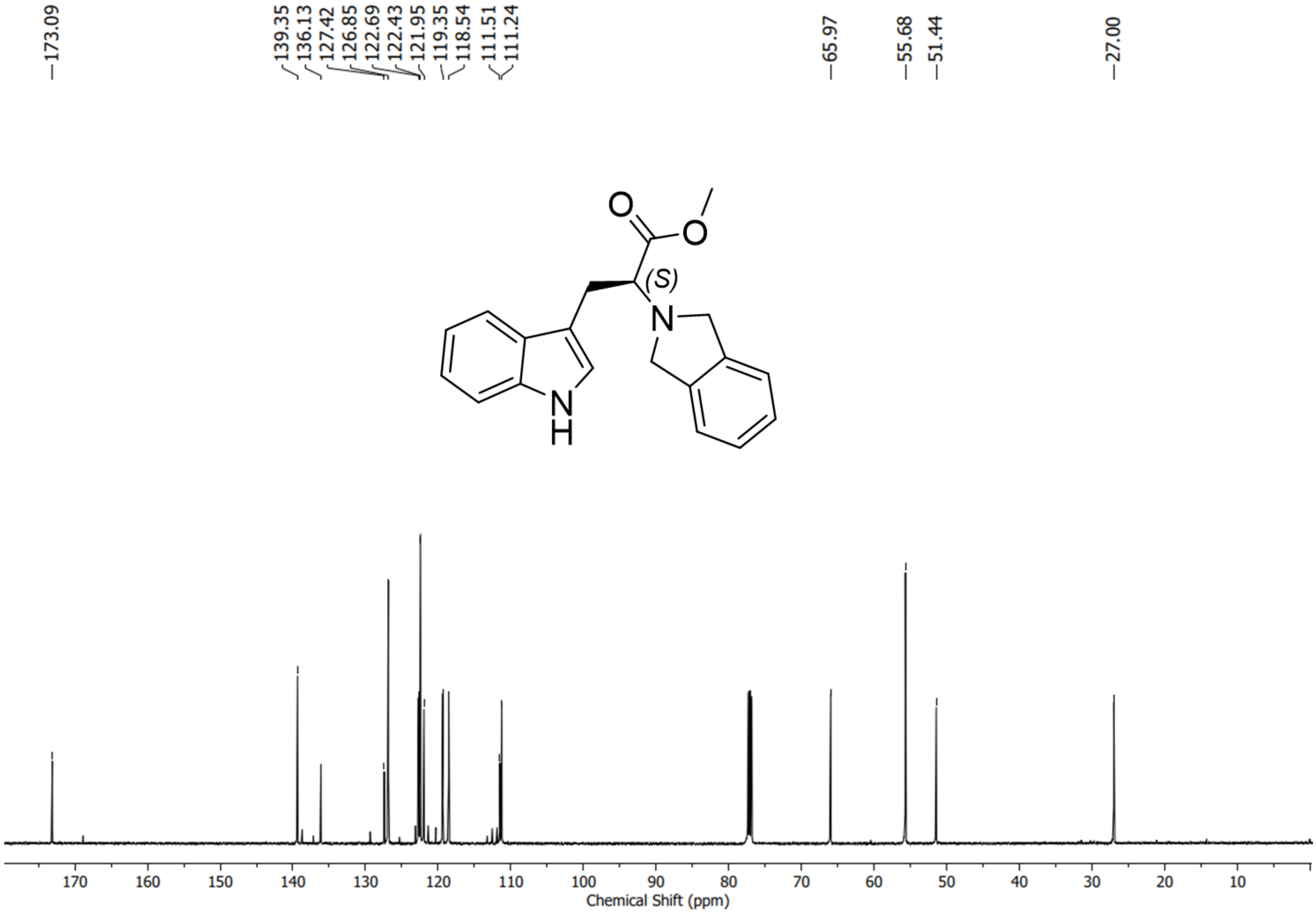
173.09
139.35
136.13
127.42
126.85
122.69
122.43
121.95
119.35
118.54
111.51
111.24
65.97
55.68
51.44
27.00
O
O
(S)
N
N
H
170
160
150
140
130
120
110
100
90
80
70
60
50
40
30
20
10
Chemical Shift (ppm)

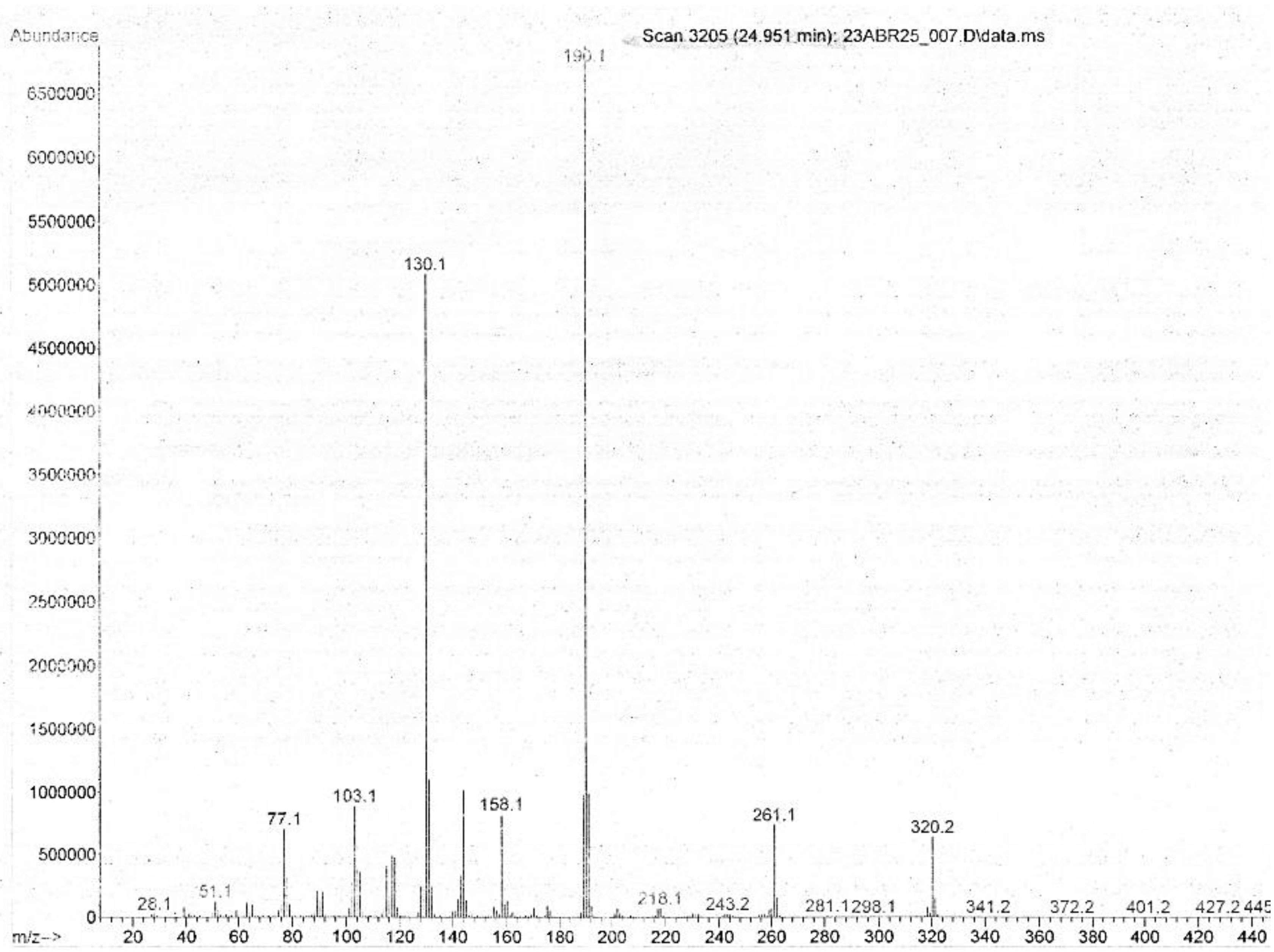
Abundance
Scan 3205 (24.951 min): 23ABR25_007.D\data.ms
m/z-->
190.1
130.1
103.1
77.1
158.1
261.1
320.2
28.1
51.1
218.1
243.2
281.1
298.1
341.2
372.2
401.2
427.2

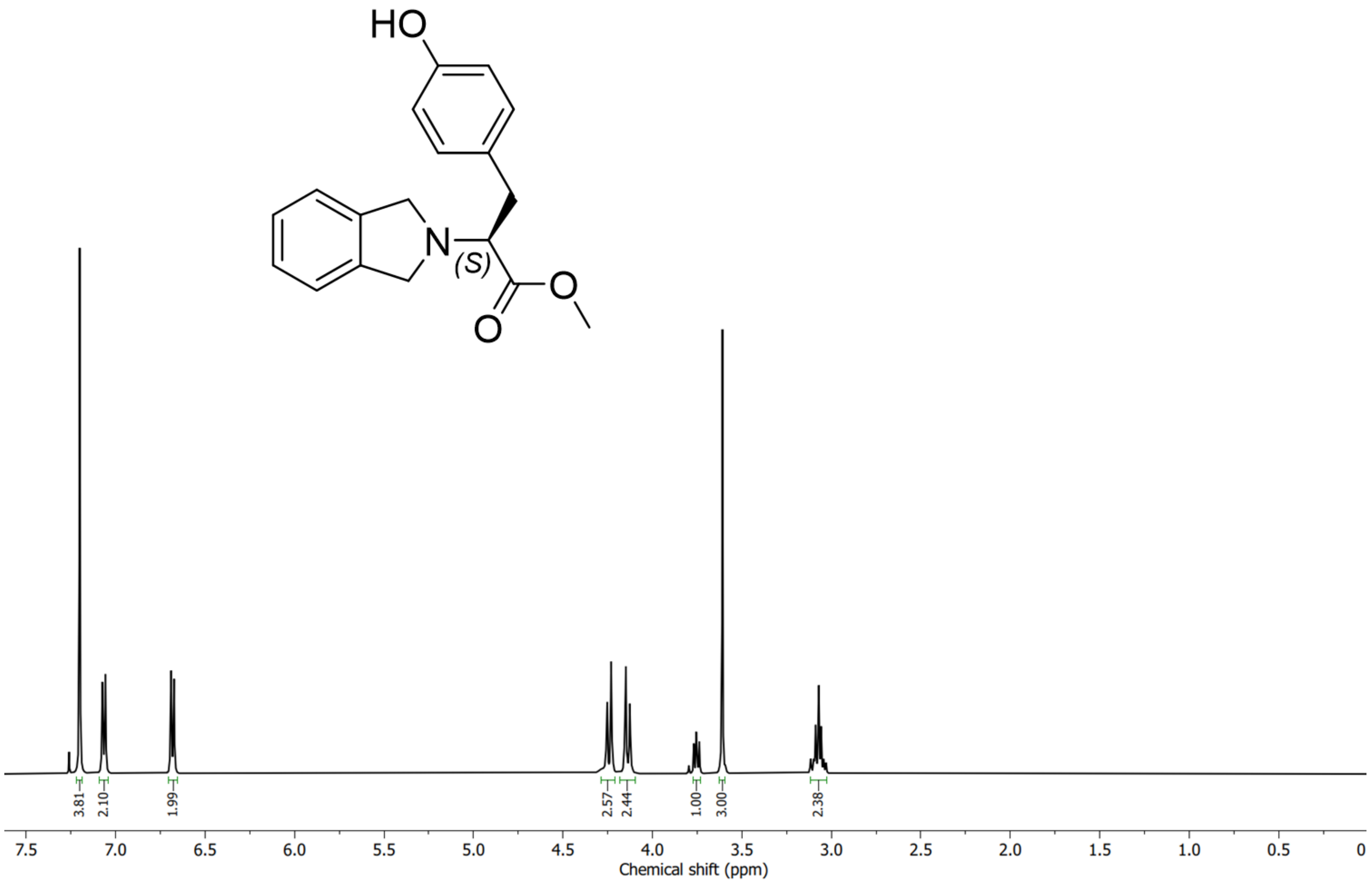

HO
N
(S)
O
O
3.81
2.10
1.99
2.57
2.44
1.00
3.00
2.38
7.5
7.0
6.5
6.0
5.5
5.0
4.5
4.0
3.5
3.0
2.5
2.0
1.5
1.0
0.5
0
Chemical shift (ppm)

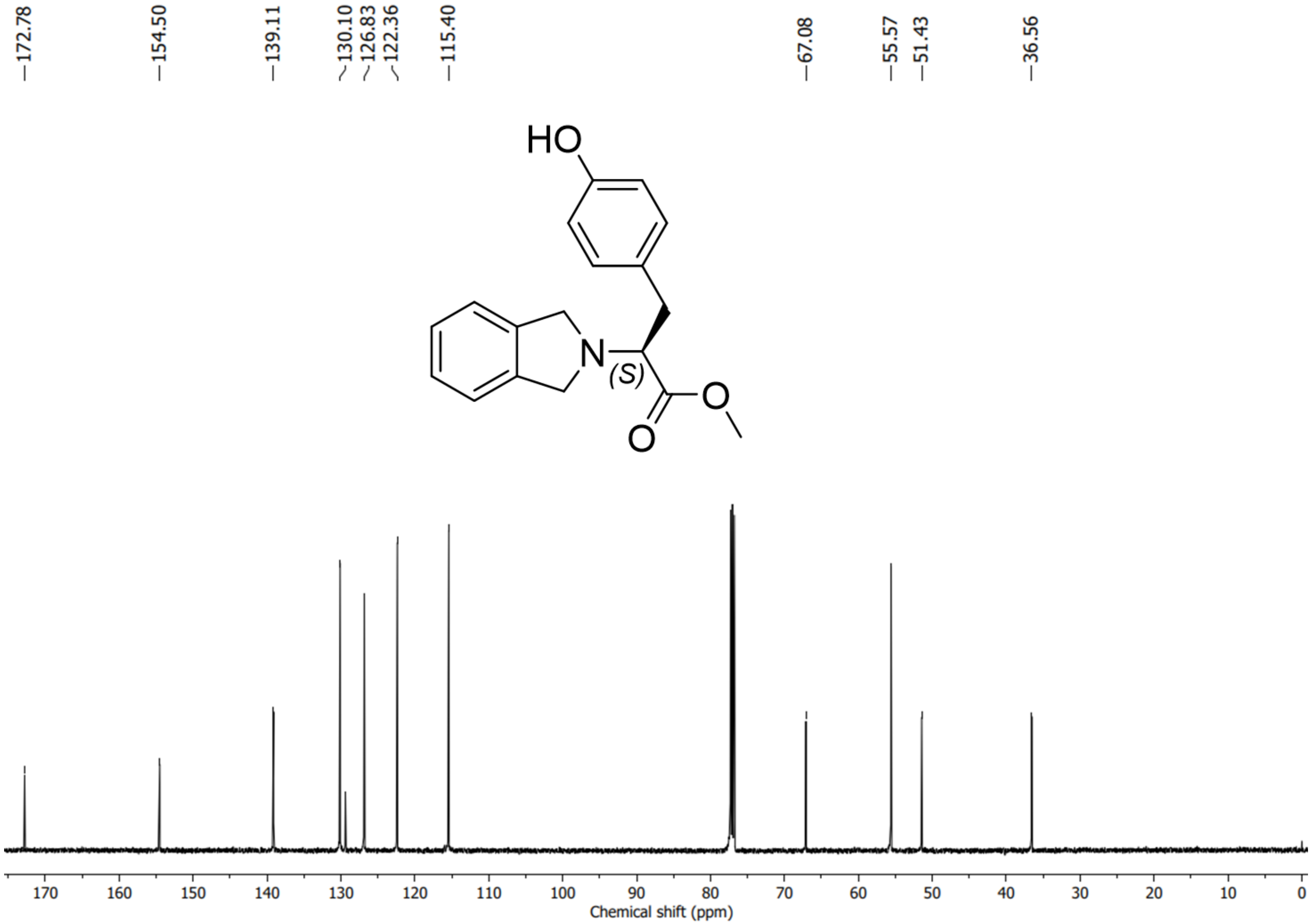
172.78
154.50
139.11
130.10
126.83
122.36
115.40
67.08
55.57
51.43
36.56
HO
N
(S)
O
O
170
160
150
140
130
120
110
100
90
80
70
60
50
40
30
20
10
0
Chemical shift (ppm)

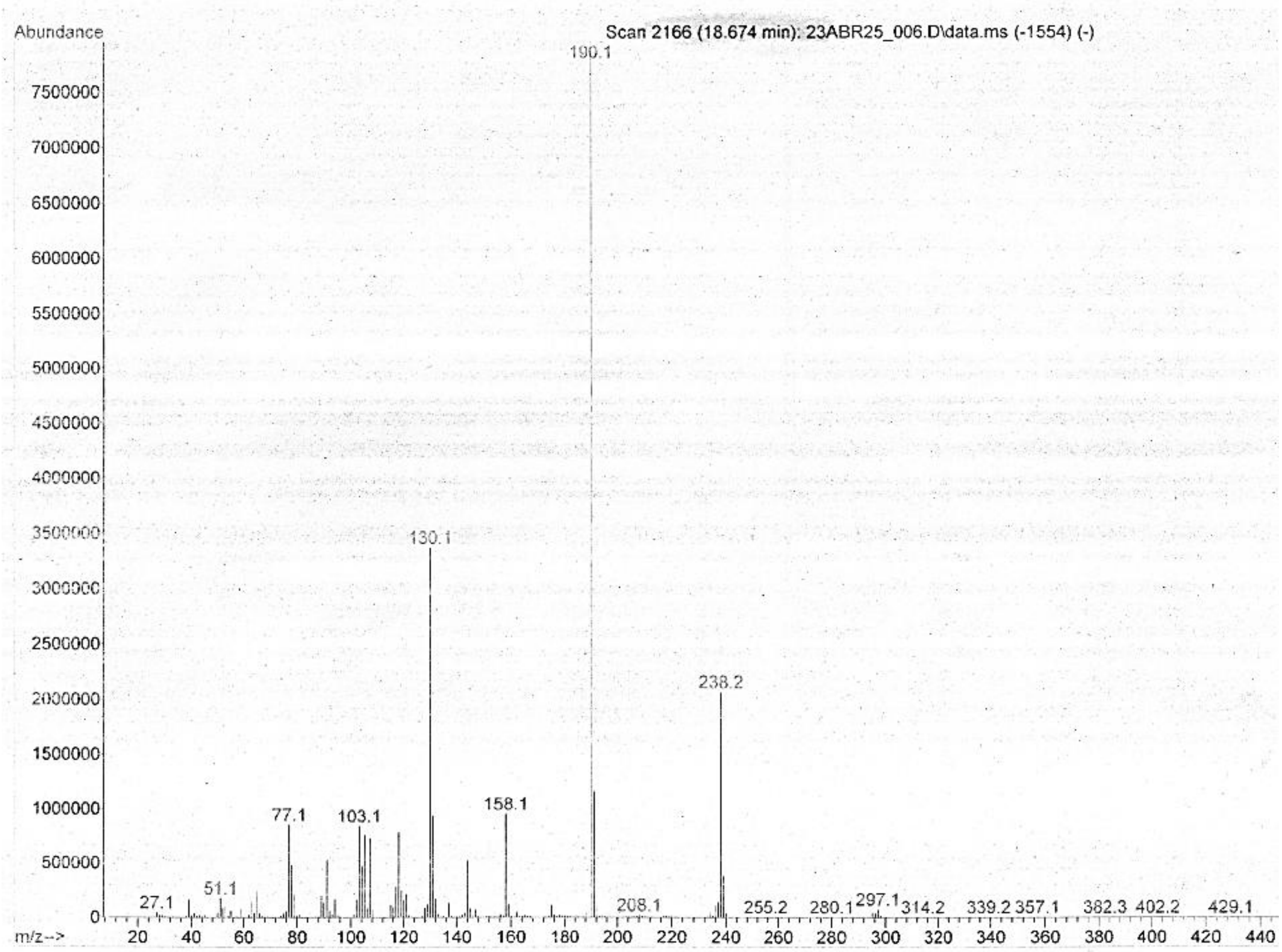
Scan 2166 (18.674 min): 23ABR25_006.D\data.ms (-1554) (-)
Abundance
7500000
7000000
6500000
6000000
5500000
5000000
4500000
4000000
3500000
3000000
2500000
2000000
1500000
1000000
500000
0
m/z-->
20 40 60 80 100 120 140 160 180 200 220 240 260 280 300 320 340 360 380 400 420 440
190.1
130.1
238.2
158.1
77.1
103.1
51.1
27.1
208.1
255.2
280.1
297.1
314.2
339.2
357.1
382.3
402.2
429.1

8 References

1. Mussulini, B. H.; Leite, C. E.; Zenki, K. C.; Moro, L.; Baggio, S.; Rico, E. P.; Rosemberg, D. B.; Dias, R. D.; Souza, T. M.; Calcagnotto, M. E.; Campos, M. M.; Battastini, A. M.; de Oliveira, D. L., Seizures induced by pentylenetetrazole in the adult zebrafish: a detailed behavioral characterization. *PLoS One* **2013,** 8, (1), e54515.
2. Zhu, S.; Noviello, C. M.; Teng, J.; Walsh, R. M., Jr.; Kim, J. J.; Hibbs, R. E., Structure of a human synaptic GABA(A) receptor. *Nature* **2018,** 559, (7712), 67-72.
3. Morris, G. M.; Huey, R.; Lindstrom, W.; Sanner, M. F.; Belew, R. K.; Goodsell, D. S.; Olson, A. J., AutoDock4 and AutoDockTools4: Automated docking with selective receptor flexibility. *J Comput Chem* **2009,** 30, (16), 2785-91.
4. Dong, J.; Wang, N. N.; Yao, Z. J.; Zhang, L.; Cheng, Y.; Ouyang, D.; Lu, A. P.; Cao, D. S., ADMETlab: a platform for systematic ADMET evaluation based on a comprehensively collected ADMET database. *J Cheminform* **2018,** 10, (1), 29.
5. Xiong, G.; Wu, Z.; Yi, J.; Fu, L.; Yang, Z.; Hsieh, C.; Yin, M.; Zeng, X.; Wu, C.; Lu, A.; Chen, X.; Hou, T.; Cao, D., ADMETlab 2.0: an integrated online platform for accurate and comprehensive predictions of ADMET properties. *Nucleic Acids Res* **2021,** 49, (W1), W5-W14.
